# Mental health conditions, physical functioning, and health-related quality of life in adults with a skeletal dysplasia: a cross-sectional multinational study

**DOI:** 10.1186/s13023-025-03610-w

**Published:** 2025-03-11

**Authors:** Elisabeth Fagereng, Su Htwe, Sam McDonald, Chloe Derocher, Marta Bertoli, Erin Carter, Anne-Mette Bredahl, Taran Blakstvedt, Micheal Wright, Cathleen Raggio, Svein Fredwall

**Affiliations:** 1 TRS National Resource Centre for Rare Disorders, Sunnaas Rehabilitation Hospital, Bjørnemyr, Norway; 2 Hospital for Special Surgery, New York, NY USA; 3 The Newcastle Upon Tyne Hospitals NHS Foundation Trust, Newcastle Upon Tyne, UK; 4 The John Walton Muscular Dystrophy Research Centre, Newcastle University, Newcastle Upon Tyne, UK

**Keywords:** Skeletal dysplasia, Mental health, Depression, Anxiety, Pain, Patient-reported outcome measures

## Abstract

**Background:**

This cross-sectional study investigated mental health conditions, physical functioning, and health-related quality of life (HRQOL) in adults with short-statured skeletal dysplasia conditions across three centres; in New York, Newcastle-upon-Tyne and Norway.

**Methods:**

Questionnaires were sent to patients registered at the centres or distributed to adults attending clinics. The questionnaires included demographics, medical history, depression (PHQ-8), anxiety (GAD-7), pain catastrophizing, activities of daily living (HAQ), and HRQOL (SF 36/RAND-36 and PROMIS-29).

**Results:**

Of the 142 participants, 62 (44%) had achondroplasia (n = 59) or hypochondroplasia (n = 3), and 80 (56%) had other skeletal dysplasia conditions (OSD), the largest groups being multiple epiphyseal dysplasia (n = 14), diastrophic dysplasia (n = 9), spondyloepiphyseal dysplasia congenita (n = 9) and pseudoachondroplasia (n = 8). Mean age was 41 (range 18–80) years. A prior psychiatric diagnosis was reported by 36%. Clinically significant symptoms of depression (PHQ-8 score ≥ 10) and anxiety (GAD-7 score ≥ 10) were reported by 23% and 13%. Almost all (99%) reported pain, while 9% had clinically significant levels of pain catastrophizing. For daily activities, the most affected domains were activities, reach and walking. The prevalence of current depression and anxiety symptoms was considerably higher in the study population than in the general US population. Participants with OSD reported more psychiatric diagnoses, depression and anxiety symptoms, more pain and challenges in performing daily activities, and lower HRQOL compared to participants with achondroplasia/hypochondroplasia.

**Conclusion:**

Adults with skeletal dysplasia appear to have an increased risk for mental health issues and reduced physical functioning, which may impact HRQOL. These findings underscore the importance of including a formal assessment of mental health, pain and daily activities as part of regular medical follow-up across the lifespan in these patients.

## Background

Skeletal dysplasia conditions are a heterogeneous group of more than 700 genetic disorders characterized by abnormal growth and remodelling of cartilage and bone [1]. The estimated prevalence of these conditions is 1 out of every 4000–5000 births. About 6% of newborns have a congenital disorder [2], with skeletal dysplasia conditions representing roughly 0.3% of these [3]. These heterogeneous disorders can affect the size and shape of the skull, trunk, and extremities to varying degrees, and are frequently associated with disproportionate short stature [3]. There is wide phenotypic variation in this population. Affected individuals are predisposed to develop orthopaedic problems such as degenerative joint disease, limb deformities, scoliosis and spinal stenosis [4], that can affect their ability to carry out tasks and activities of daily living. A high prevalence of chronic pain has been reported in skeletal dysplasia populations [5, 6]. Short stature and chronic pain can reduce physical function and pose barriers to navigating physical spaces designed for average-statured bodies. Living with the scrutiny of society as a short person may also contribute as a stressor. All of these factors can affect the well-being of the individual living with a skeletal dysplasia and may affect quality of life for this population.

The literature seems to agree that people with a skeletal dysplasia have reduced physical abilities in comparison to the general population [4–7], but the conclusions regarding mental health are mixed. In 2010, Shakespeare et al. reported on 87 adults with skeletal dysplasia in the north of England (over half having achondroplasia), of whom 37% had experienced mental health issues, predominantly depression [8]. Ten years later, a study conducted in the Unites States (US) explored the prevalence of mental health conditions and pain in a cohort of 336 adults with self-reported skeletal dysplasia diagnoses (over half of whom had achondroplasia) [6]. Depressive symptoms or a prior diagnosis of depression was noted by 34% of the study participants, and 31% noted symptoms of anxiety or had a previous diagnosis of anxiety. Pain was reported by 75% of respondents, and severe pain that interfered with daily life functioning was associated with the presence of depression and anxiety [6].

In this study, we aimed to investigate whether these findings are representative for a broader skeletal dysplasia population across the US, United Kingdom (UK), and Norway. We explored the prevalence and symptom severity of mental health conditions, physical functioning, pain, and health-related quality of life (HRQOL) by using well-recognized generic questionnaires, including specific instruments for assessing depression and anxiety, to compare results to the general population and across skeletal dysplasia groups.

## Methods

This cross-sectional multinational study was conducted in three centres; Hospital for Special Surgery in New York (US), the Northern Genetics Service in Newcastle-upon-Tyne (UK), and TRS National Resource Centre for Rare Disorders (Norway). Data collection was conducted from March 2020 to August 2024.

### Participants and recruitment

Participants were eligible for the study if they were aged 18 years or older and had a confirmed clinical or genetic diagnosis of short-statured skeletal dysplasia. Adults at Hospital for Special Surgery (US) were recruited during clinic visits or through an invitation letter by post. Participants from Newcastle-upon-Tyne (UK) were identified within the cohort of patients seen in the Bone Genetic Service of the Northern Genetic Service and were invited during a clinic visit or by post. In addition, the patient advocacy groups Beacon for Rare Disease and The Restricted Growth Association circulated a link to participation on social media platforms, including Twitter and Facebook, to reach patients outside of the Newcastle-upon-Tyne area. In Norway, an invitation letter was sent to all adults aged 18 years or older registered with a short-statured skeletal dysplasia at the National Resource Centre for Rare Disorders. In addition, the study was announced on the website and social media of the resource centre and The Norwegian Restricted Growth Association (NiK), using text and a short video for recruitment.

### Measurements

#### Demographic and medical variables

Participant characteristics included sex, age, employment status, years of education and annual income. Medical variables included skeletal dysplasia diagnosis, surgical history, pain location and intensity, and history of psychiatric illness.

#### Depression and anxiety

The Patient Health Questionnaire (PHQ-8) depression module measured depressive symptoms. The PHQ-8 covers the first eight diagnostic criteria for depressive disorder in the Diagnostic and Statistical Manual of Mental Disorders (DSM-IV) [9]. A score of 10 or more indicates clinically significant depressive symptoms [9]. The US [10], UK [11] and Norwegian version [12] of PHQ-8 have shown good psychometric properties and is regarded as a reliable and valid measure of depression severity, commonly used in clinical practice and research.

The 7-item Generalized Anxiety Disorder (GAD-7) questionnaire measured anxiety symptoms [13]. A score of 10 or more is accepted as a threshold for clinically significant anxiety symptoms [13]. The GAD-7 has been validated as an anxiety screening tool and severity measure in different populations [14, 15].

#### Pain

In addition to exploring pain prevalence, location, and intensity, we used the Pain Catastrophizing Scale. The Pain Catastrophizing Scale is a commonly used and validated self-report questionnaire to measure the degree of pain-related catastrophic thinking and is validated in both English [16] and Norwegian [17]. The questionnaire consist of 13 items, where higher scores reflect more pain catastrophizing. A total score of 30 or more represents a clinically significant level of pain catastrophizing, corresponding to the 75th percentile of the distribution of scores in chronic pain patients [18].

#### Activities of daily living (ADL)

The Stanford Health Assessment Questionnaire Disability Index (HAQ) measured physical function and disability [19]. The HAQ consists of 20 questions in eight categories, representing a comprehensive set of functional activities: dressing and grooming; arising; eating; walking; hygiene; reach (including the items: reach and get down a 5-pound (approximately 2.25 kilo) object from just above your head, and bend down to pick up clothing from the floor); grip; and activities (including the items: run errands and shop, get in and out of car, do chores such as vacuuming and yard work) [20]. The HAQ is among the most commonly used functional measures in rheumatology and arthritis, but has also been used in a wide range of other chronic disorders [19, 20]. Each item is rated from 0 to 3 (0 = no difficulty; 1 = some difficulty; 2 = much difficulty; 3 = not able to do) [20]. A difference of 0.22–0.25 is considered a minimum clinical important difference [20]. The HAQ has been translated into both Norwegian and Swedish, which are very similar languages. The Swedish version has been validated [21].

#### Health-related quality of life (HRQOL)

We used the Medical Outcomes Study 36-item Short Form Health Survey (SF-36/RAND-36) and the Patient Reported Outcomes Measurement Information System 29 (PROMIS-29) to assess HRQOL. These are widely used self-report questionnaires measuring physical and mental wellbeing. Due to differences in licence policies, the US and UK site used the SF-36, while the Norwegian site used the RAND-36. Both questionnaires include the same items and consist of 36 items which encompass eight health domains: Physical functioning; Role limitations due to physical health; Bodily pain; General health; Vitality; Social functioning; Role limitations due to emotional health; and Mental health [22]. Each domain is scored from 0 to 100, where higher scores indicate better functioning. Furthermore, physical and mental component scores (PCS and MCS) were computed using the oblique method [23, 24]. The component scores are transformed into T-scores with a mean of 50 and standard deviation of 10. The SF-36/RAND-36 have been validated in both the US [22], UK [25] and Norway [26].

The PROMIS-29 has become a widely used instrument internationally to assess aspects of physical, mental, and social health in different patient populations [27, 28]. The PROMIS-29 includes seven health domains: Physical function; Pain interference; Fatigue; Sleep disturbance; Depression; Anxiety; and Ability to participate in social roles and activities [29]. Each domain comprises of four items. Higher scores indicate more of the concept being measured, i.e. a higher score in Physical function represents better physical health, whereas a higher score in Depression represents higher depressive symptom burden. Physical and mental health component scores (PCS and MCS) were calculated according to the description provided by Hays et al. [29]. Both domain scores and component scores are presented as T-scores. The PROMIS-29 has shown to be a valid measure with good psychometric properties [29–31].

### Statistical analyses

Descriptive statistics were performed for sample characteristics and outcome through absolute numbers (n) with percentages (%) and mean with standard deviation (SD). Comparison between the study population and general population was performed with one-way t-test. Furthermore, the study population was categorized into two groups: “Achondroplasia/hypochondroplasia” and “Other skeletal dysplasias”. According to the Nosology of genetic skeletal disorders, achondroplasia and hypochondroplasia both belong to the FGFR3-related chondrodysplasias [1]. Due to few respondents with hypochondroplasia in this study, participants with achondroplasia and hypochondroplasia therefore were grouped together. The group of other skeletal dysplasia conditions consisted of 22 different skeletal dysplasia diagnoses belonging to several different groups of genetic skeletal disorders [1]. For descriptive and comparative analyses, these were grouped together due to the low number of participants within each diagnosis. Differences between adults with achondroplasia/hypochondroplasia and adults with other skeletal dysplasia conditions were analysed with independent samples t-test. Results were provided as mean differences with 95% confidence interval (CI). Missing data on questionnaires were handled by imputing the missed item with the mean. Statistical analyses were carried out using SPSS 29.0 (IBM Inc., Armonk, NY, USA). Statistical significance was defined as an alpha-level of *p* ≤ 0.05.

## Results

### Demographic and clinical variables

Overall, 142 adults with a short-statured skeletal dysplasia participated in the study: achondroplasia (n = 59); multiple epiphyseal dysplasia (n = 14); diastrophic dysplasia (n = 9); spondyloepiphyseal dysplasia (SED) congenita (n = 9); pseudoachondroplasia (n = 8); SED (n = 7); trichorhinophalangeal syndrome 1 and 3 (n = 4); mucopolysaccharidosis IV (n = 4); hypochondroplasia (n = 3); spondyloepimetaphyseal dysplasia (n = 3); Schmid metaphyseal chondrodysplasia (n = 3); Kniest dysplasia (n = 3); acrodysostosis (n = 2); brachyolmia (n = 2); SED Tarda (n = 2); ACAN related short stature (n = 2); cartilage-hair hypoplasia (n = 2); chondrodysplasia (n = 2); Steel syndrome (n = 1); metatrophic dysplasia (n = 1); melorheostosis (n = 1); and femoral facial syndrome (n = 1).

**Fig. 1:**
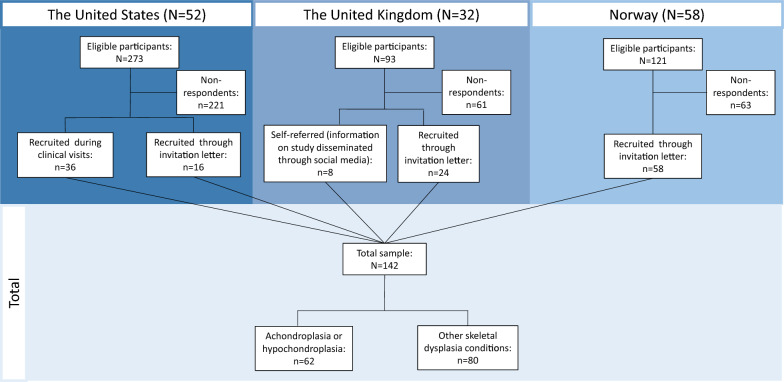
Flowchart showing inclusion of study participants

Of those invited to participate in this study, the response rates were as follows; 52 out of 273 (19%) in the US study population, 32 out of 93 (34%) in the UK study population, and 58 out of 121 (48%) in Norwegian study population, see Fig. 1. Mean age was 41.3 years (range 18–80 years), 59% were female, 59% had completed higher education, and 42% worked full-time (Table 1). The distribution for sex and age was fairly similar between the three countries, but respondents from the UK had a lower education level and a higher proportion in the lowest income category. More than half of the US respondents were in the highest income category (annual income > $60 000).

**Table 1:** Participant characteristics

	Total sample (N = 142)	United States (n = 52)	United Kingdom (n = 32)	Norway (n = 58)
	n (%)	n (%)	n (%)	n (%)
Females Males	83 (59%) 59 (41%)	31 (60%) 21 (40%)	19 (59%) 13 (41%)	33 (57%) 25 (43%)
Age, year, mean (range)	41.3 (18–80)	43.3 (18–80)	39.6 (21–72)	40.4 (18–73)
Education level				
Primary	9 (6%)	3 (6%)	3 (9%)	3 (5%)
Upper secondary	46 (32%)	12 (23%)	11 (34%)	23 (40%)
Higher education	83 (59%)	36 (69%)	15 (47%)	32 (55%)
Employment status				
Full time	59 (42%)	27 (52%)	13 (41%)	19 (33%)
Part time	26 (18%)	5 (10%)	10 (31%)	11 (19%)
Full disability benefit	10 (7%)	6 (12%)	–	4 (7%)
Other	47 (33%)	14 (27%)	9 (28%)	24 (41%)
Income a year				
Less than $20 000	41 (29%)	14 (27%)	15 (47%)	12 (21%)
$20 000–$60 000	54 (38%)	9 (17%)	12 (38%)	33 (57%)
More than $60 001	43 (30%)	29 (56%)	4 (13%)	10 (17%)
Skeletal dysplasia diagnosis				
ACH or HCH^a^	62 (44%)	16 (31%)	13 (41%)	33 (57%)
Other skeletal dysplasias^b^	80 (56%)	36 (69%)	19 (59%)	25 (43%)
Genetically confirmed	88 (62%)	31 (60%)	17 (53%)	40 (69%)
Surgical burden				
Total number of surgeries, *mean (range)*	7.1 (0–36)	9.5 (0–36)	6 (0–20)	5.7 (0–24)
Type of surgery				
Spine	45 (32%)	21 (40%)	4 (13%)	20 (35%)
Ear	36 (25%)	13 (25%)	7 (22%)	16 (28%)
Tonsillectomy	39 (28%)	12 (23%)	8 (25%)	19 (33%)
Foot	34 (24%)	14 (27%)	4 (13%)	15 (26%)
Hand	7 (5%)	2 (4%)	2 (6%)	3 (5%)
Joint replacement	43 (30%)	19 (37%)	9 (28%)	14 (24%)
Intramedullary rodding	10 (7%)	8 (15%)	2 (6%)	–
Leg realignment	49 (35%)	19 (37%)	12 (38%)	18 (31%)
Leg lengthening	11 (8%)	1 (2%)	1 (3%)	9 (16%)
Other upper extremity	22 (16%)	9 (17%)	9 (28%)	4 (7%)
Other lower extremity	34 (24%)	20 (39%)	6 (19%)	8 (14%)

^a^ACH: achondroplasia; HCH: hypochondroplasia

^b^Other skeletal dysplasia conditions: ACAN related short stature; acrodysostosis; brachyolmia; cartilage-hair hypoplasia; chondrodysplasia; diastrophic dysplasia; femoral facial syndrome; Kniest dysplasia; melorheostosis; metatrophic dysplasia; mucopolysaccharidosis IV (Morquio syndrome); multiple epihyseal dysplasia (MED); pseudoachondroplasia; Schmid metaphyseal chondrodysplasia; SED congenita (SEDC); SED tarda; spondyloepimetaphyseal dysplasia (SEMD); spondyloepiphyseal dysplasia (SED); Steel syndrome; trichorhinophalangeal syndrome (TRPS) 1 and 3

Achondroplasia/hypochondroplasia comprised the largest skeletal dysplasia subgroup (44%). The majority (62%) of all study participants had a molecular genetic confirmation of their diagnosis. Mean number of surgeries per participant was 7.1, ranging from 0 to 36. Leg realignment was the most prevalent type of surgery (35%), followed by spine surgery (32%) and joint replacement (30%). A detailed overview of types of surgery for each diagnosis is provided in Table 2.

**Table 2:** Overview of types of surgery for each diagnosis

	N (%)	Spine	Ear	Tonsill- ectomy	Foot	Hand	Joint replacement	Rodding	Leg realignment	Leg lengthening	Other upper extremity	Other lower extremity
		n (%)	n (%)	n (%)	n (%)	n (%)	n (%)	n (%)	n (%)	n (%)	n (%)	n (%)
Achondroplasia	59 (41.5)	26 (44.1)	24 (40.7)	30 (50.8)	7 (11.9)	1 (1.7)	4 (6.8)	2 (3.4)	22 (37.3)	9 (15.3)	8 (13.6)	7 (11.9)
MED^a^	14 (9.9)	–	–	2 (14.3)	4 (28.6)	1 (7.1)	9 (64.3)	–	5 (35.7)	–	2 (14.3)	2 (14.3)
Diastrophic dysplasia	9 (6.3)	4 (44.4)	–	–	8 (88.9)	1 (11.1)	3 (33.3)	3 (33.3)	2 (22.2)	–	1 (11.1)	3 (33.3)
SED^b^ congenita	9 (6.3)	2 (22.2)	2 (22.2)	1 (11.1)	2 (22.2)	1 (11.1)	5 (55.6)	–	2 (22.2)	–	3 (33.3)	3 (33.3)
Pseudoachondroplasia	8 (5.6)	3 (37.5)	–	–	3 (37.5)	1 (12.5)	6 (75)	–	5 (62.5)	–	–	2 (25)
SED	7 (4.9)	1 (14.3)	2 (28.6)	1 (14.3)	3 (42.9)	–	4 (57.1)	1 (14.3)	1 (14.3)	1 (14.3)	3 (42.9)	3 (42.9)
TRPS^c^ 1 and 3	4 (2.8)	–	2 (50)	1 (25)	2 (50)	–	1 (25)	–	1 (25)	–	–	–
Morquio syndrome^d^	4 (2.8)	3 (75)	–	1 (25)	–	1 (25)	1 (25)	–	1 (25)	–	–	3 (75)
Hypochondroplasia	3 (2.1)	1 (33.3)	–	–	–	–	–	–	–	1 (33.3)	–	–
SEMD^e^	3 (2.1)	1 (33.3)	–	1 (33.3)	–	–	1 (33.3)	2 (66.7)	2 (66.7)	–	1 (33.3)	–
Schmid metaphyseal chondrodysplasia	3 (2.1)	–	–	–	–	–	1 (33.3)	1 (33.3)	1 (33.3)	–	–	2 (66.7)
Kniest dysplasia	3 (2.1)	–	3 (100)	–	–	1 (33.3)	2 (66.7)	–	2 (66.7)	–	–	2 (66.7)
Acrodysostosis	2 (1.4)	–	–	–	1 (50)	–	1 (50)	–	–	–	1 (50)	1 (50)
Brachyolmia	2 (1.4)	–	–	–	–	–	1 (50)	–	1 (50)	–	–	–
SED tarda	2 (1.4)	–	–	–	1 (50)	–	2 (100)	–	–	–	–	–
ACAN related short stature	2 (1.4)	–	–	–	–	–	–	–	–	–	–	–
Cartilage-hair hypoplasia	2 (1.4)	–	1 (50)	–	–	–	–	–	1 (50)	–	–	2 (100)
Chondrodysplasia	2 (1.4)	2 (100)	–	–	–	–	–	–	–	–	–	1 (50)
Steel syndrome	1 (0.7)	–	1 (100)	–	1 (100)	–	–	–	1 (100)	–	–	1 (100)
Metatrophic dysplasia	1 (0.7)	1 (100)	–	–	–	–	–	–	1 (100)	–	–	1 (100)
Melorheostosis	1 (0.7)	1 (100)	–	1 (100)	1 (100)	–	1 (100)	1 (100)	–	–	1 (100)	1 (100)
Femoral facial syndrome	1 (0.7)	–	1 (100)	1 (100)	1 (100)	–	1 (100)	–	1 (100)	–	–	1 (100)

^a^MED: multiple epihyseal dysplasia

^b^SED: spondyloepiphyseal dysplasia

^c^TRPS: trichorhinophalangeal syndrome

^d^Morquio syndrome: mucopolysaccharidosis IV

^e^SEMD: spondyloepimetaphyseal dysplasia

### Mental health conditions

#### Skeletal dysplasia study population versus general population

**Fig. 2:**
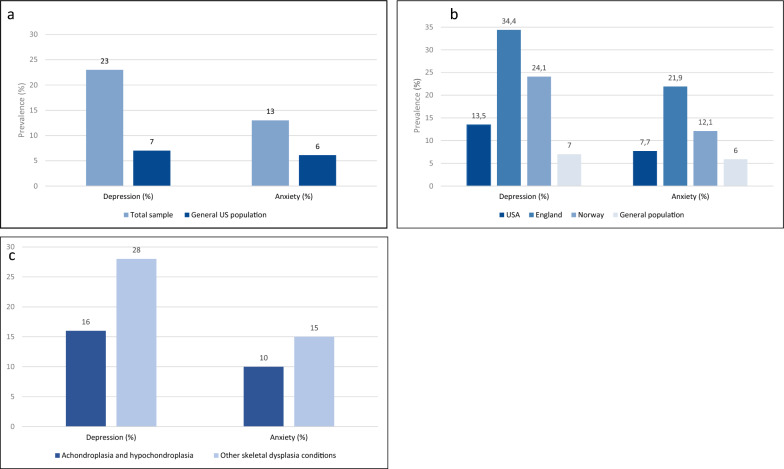
Prevalence of current clinically significant symptoms of depression and anxiety, as measured by PHQ-8 score ≥ 10 and GAD-7 score ≥ 10. **a:** The prevalence in the total study population compared to the general US population. **b:** Prevalence in the US, UK and Norwegian study population and the general population. **c:** The prevalence in achondroplasia/hypochondroplasia compared to other skeletal dysplasia conditions

A history of a psychiatric diagnosis was reported by 36% of the total study population. Anxiety (23%) and depression (22%) were the most prevalent mental health conditions reported (Table 3). Clinically significant levels of current depressive symptoms (PHQ-8 score ≥ 10) were reported in 23% of the total study population, compared to 7% in the US general population [32]. Current clinically significant symptom levels of anxiety (GAD-7 ≥ 10) were reported in 13% of the total study population, compared to 6% in the US population (Fig. 2a) [33].

**Table 3:** Mental health conditions in the total study population and skeletal dysplasia subgroups

	Total sample (N = 142)	ACH and HCH (n = 62)	OSD (n = 80)
	n (%)	n (%)	n (%)
History of psychiatric diagnosis	51 (36%)	19 (31%)	32 (40%)
Anxiety	33 (23%)	10 (16%)	23 (29%)
Depression	31 (22%)	11 (18%)	20 (25%)
ADHD	9 (6%)	4 (7%)	5 (6%)
Bipolar disorder	3 (2%)	–	3 (4%)
OCD	3 (2%)	1 (2%)	2 (3%)
PTSD	3 (2%)	–	3 (4%)
Substance abuse	2 (1%)	1 (2%)	1 (1%)
Eating disorder	1 (1%)	–	1 (1%)
Other	5 (4%)	2 (3%)	3 (4%)
Clinical depressive symptoms (PHQ-8 ≥ 10)	32 (23%)	10 (16%)	22 (28%)
Clinical anxiety symptoms (GAD-7 ≥ 10)	18 (13%)	6 (10%)	12 (15%)
History of depression (n = 31) and current clinical depressive symptoms	18/31 (58%)	6 (10%)	11 (14%)
History of anxiety (n = 33) and current clinical anxiety symptoms	9/33 (27%)	3 (5%)	6 (8%)

ACH, achondroplasia; ADHD, Attention-Deficit/Hyperactivity Disorder; GAD-7, Generalized Anxiety Disorder 7; HCH, hypochondroplasia; OCD, Obsessive–Compulsive Disorder; OSD, other skeletal dysplasia conditions; PHQ-8, Patient Health Questionnaire 8; PTSD, Posttraumatic Stress Disorder

Of those with a previous history of depression, 18/31 (58%) reported current clinically significant symptoms of depression. Of those with a previous history of anxiety, 9/33 (27%) reported current clinically significant symptoms of anxiety. Respondents from the UK had the highest prevalence rates of both current clinically significant anxiety and depression, while the respondents from the US had the lowest prevalence rates (Fig. 2b).

#### Achondroplasia/hypochondroplasia vs. other skeletal dysplasia conditions

Overall, participants with other skeletal dysplasia conditions had a higher prevalence of previous history of mental health conditions (40% vs. 31%), in particular anxiety (29% vs. 16%) and depression (25% vs. 18%) (Table 3). The prevalence of current clinically significant symptoms of depression and anxiety were also higher among participants with other skeletal dysplasia conditions compared to the achondroplasia/hypochondroplasia group. Current depressive symptoms (PHQ-8 score ≥ 10) were found in 28% in the other skeletal dysplasia population vs. 16% in achondroplasia/hypochondroplasia, while current anxiety symptoms (GAD-7 ≥ 10) were found in 15% vs. 10%, respectively (Fig. 2c).

### Physical functioning

#### Skeletal dysplasia study population vs. general population

All participants except for one (99%) reported pain in at least one body site. The most frequent pain site locations were lower back (63%), lower extremities (58%), and neck (31%) (Table 4). Mean pain intensity (NRS scale 0–10) was 6.0. The prevalence of clinically significant levels of pain catastrophizing (total score ≥ 30) was 9% in the total study population.

**Table 4:** Physical functioning

	Total sample (N = 142)	ACH and HCH (n = 62)	OSD (n = 80)
	n (%)	n (%)	n (%)
Pain			
Prevalence	141 (99%)	62 (100%)	79 (99%)
Pain site location			
Lower back	89 (63%)	39 (63%)	50 (63%)
Lower extremities	82 (58%)	26 (42%)	56 (70%)
Neck	44 (31%)	14 (23%)	30 (38%)
Upper extremities	31 (22%)	11 (18%)	20 (25%)
Upper back	25 (18%)	8 (13%)	17 (21%)
Headache	22 (16%)	9 (15%)	13 (16%)
Intensity NRS 1–10, m*ean (SD)*	6.0 (2.4)	5.8 (2.4)	6.3 (2.4)
PCS, above cutoff (≥ 30)	12 (9%)	2 (3%)	10 (13%)

	Mean (SD)	Mean (SD)	Mean (SD)
Activities of daily living (ADL)			
HAQ total mean score	0.85 (0.70)	0.76 (0.70)	0.92 (0.69)
HAQ Category sum score			
Dressing and grooming	0.75 (0.86)	0.72 (0.92)	0.77 (0.82)
Arising	0.66 (0.76)	0.62 (0.80)	0.70 (0.74)
Eating	0.56 (0.84)	0.41 (0.69)	0.68 (0.93)
Walking	1.02 (0.99)	0.90 (0.98)	1.11 (1.00)
Hygiene	0.88 (0.97)	0.88 (0.92)	0.87 (1.00)
Reach	1.04 (0.98)	0.82 (0.97)	1.22 (0.96)
Grip	0.54 (0.87)	0.43 (0.83)	0.63 (0.89)
Activities	1.33 (1.01)	1.26 (1.10)	1.38 (0.94)

Total study population and skeletal dysplasia subgroups

ACH, achondroplasia; HAQ, Health Assessment Questionnaire; HCH, hypochondroplasia; OSD, other skeletal dysplasia conditions; PCS, Pain Catastrophizing Scale

Regarding ability to perform activities of daily living (ADL), mean (SD) HAQ score in the total study population was 0.85 (0.70). The most affected domains were *activities* (mean score 1.33), *reach* (mean score 1.04), and *walking* (mean score 1.02) (Table 4).

#### Achondroplasia/hypochondroplasia vs. other skeletal dysplasia conditions

Approximately 63% of the participants in each group reported pain in the lower back region (Table 4). Of study participants with other skeletal dysplasia conditions, 70% reported pain in the lower extremities, compared to 42% in the achondroplasia/hypochondroplasia group. Mean pain intensity was higher in participants with other skeletal dysplasias conditions compared to participants with achondroplasia/hypochondroplasia (NRS mean score 6.3 vs 5.8). Clinically significant levels of pain catastrophizing were found in 3% of participants with achondroplasia/hypochondroplasia compared to 13% in participants with other skeletal dysplasia conditions.

Participants with other skeletal dysplasia conditions reported more challenges (higher scores) in performing activities of daily living (ADL) in all eight subcategories except for hygiene, and in total mean HAQ. The subcategories reflecting the greatest differences between the two groups were *reach* (mean difference 0.40), *eating* (mean difference 0.29) and *walking* (mean difference 0.27) (Table 4).

### Health-related quality of life (HRQOL)

#### Skeletal dysplasia study population vs. general population

**Fig. 3:**
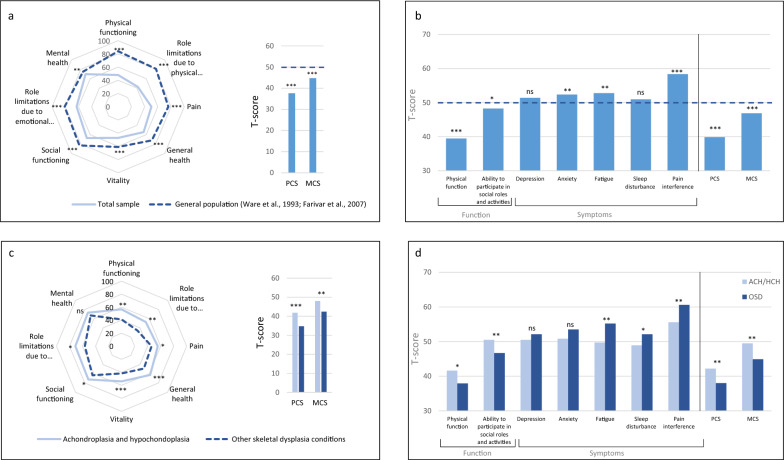
Health-related quality of life assessed by SF-26/RAND-36 and PROMIS-29. **a:** SF-36/RAND-36 domain score, and physical and mental health component scores (PCS and MCS) in the total study population compared to the general population. **b:** PROMIS-29 domain score, PCS and MCS in the total study population compared to normative mean (M = 50) reflected by the stippled orange line. **c:** SF-36/RAND-36 domain score, PCS and MCS in achondroplasia/hypochondroplasia (ACH/HCH) compared to other skeletal dysplasia conditions. **d:** PROMIS-29 domain score, PCS and MCS in ACH/HCH compared to other skeletal dysplasia conditions (OSD)

HRQOL was measured using the SF-36/RAND-36 and the PROMIS-29. For the SF-36/RAND-36, the total skeletal dysplasia study population reported significantly lower scores (reflecting reduced HRQOL) in all physical and mental health domains, including PCS (physical health component score) and MCS (mental health component score), compared to the general US population (Table 5) [24, 34]. The lowest scores among the eight domains were in the domains *Role limitations due to physical health* (mean 42.4) and *Vitality* (mean 47.2), whereas *Role limitations due to physical health* and *Physical functioning* were the two domains with the highest observed discrepancy compared to the general US population (Fig. 3a).

**Table 5:** Health related quality of life (SF/RAND-36 and PROMIS-29) for total study population and skeletal dysplasia subgroups

	Total sample (N = 142)	ACH and HCH (n = 62)	OSD (n = 80)	Mean diff	CI 95%	*p*
	Mean (SD)	Mean (SD)	Mean (SD)			
SF-36/RAND-36						
Physical functioning	48.0 (29.6)	56.7 (31.2)	41.2 (26.6)	15.5	5.7–25.4	**< 0.01**
Role limitations due to physical health	42.4 (42.4)	52.7 (43.6)	34.3 (39.9)	18.4	4.4–32.4	**0.01**
Bodily pain	50.3 (25.2)	55.6 (25.6)	46.1 (24.3)	9.5	1.2–17.9	**< 0.05**
General health	54.5 (23.7)	62.3 (20.7)	48.5 (24.1)	13.8	6.2–21.4	**< 0.001**
Vitality	47.2 (22.6)	54.0 (21.8)	41.8 (21.8)	12.2	4.9–19.6	**0.001**
Social functioning	67.1 (26.6)	72.2 (28.0)	63.1 (24.9)	9.0	0.2–17.9	**< 0.05**
Role limitations due to emotional health	62.9 (42.5)	71.0 (40.3)	56.4 (43.4)	14.6	0.4–28.7	**< 0.05**
Mental health	69.7 (19.8)	73.1 (18.5)	67.0 (20.5)	6.1	− 0.5–12.7	ns
PCS	37.6 (11.2)	41.4 (11.1)	34.6 (10.4)	6.8	3.2–10.4	**< 0.001**
MCS	44.7 (9.9)	47.6 (9.4)	42.4 (9.8)	5.2	1.9–8.5	**< 0.01**
PROMIS-29						
Physical function	39.5 (9.8)	41.6 (10.0)	37.9 (9.4)	3.7	0.5–7.0	**< 0.05**
Ability to participate in social roles and activities	48.3 (8.9)	50.5 (8.5)	46.7 (8.9)	3.8	0.9–6.7	**0.01**
Depression	51.4 (9.7)	50.5 (9.5)	52.1 (9.9)	− 1.5	− 4.8–1.7	0.36
Anxiety	52.4 (10.2)	50.8 (10.5)	53.5 (9.9)	− 2.7	− 6.2–0.7	0.12
Fatigue	52.8 (10.4)	49.7 (9.1)	55.2 (10.8)	− 5.6	− 8.9–− 2.3	**< 0.01**
Sleep disturbance	50.7 (9.1)	48.9 (8.1)	52.1 (9.6)	− 3.1	− 6.1–− 0.1	**< 0.05**
Pain interference	58.4 (10.5)	55.6 (10.4)	60.6 (10.0)	− 5.0	− 8.4–− 1.6	**< 0.01**
PCS	39.9 (10.0)	42.2 (10.1)	38.0 (9.5)	4.2	0.9–7.5	**0.01**
MCS	46.9 (8.9)	49.5 (8.0)	44.9 (9.1)	4.6	1.7–7.5	**< 0.01**

ACH, achondroplasia; CI, confidence interval; HCH, hypochondroplasia; MCS, mental health component score; OSD, other skeletal dysplasia conditions; PCS, physical health component score; PROMIS-29, Patient Reported Outcomes Measurement Information System 29; RAND-36, RAND-36 Item Health Survey; SF-36, Short Form Health Survey

For the PROMIS-29, both PCS (mean 39.9) and MCS (mean 46.9) were significantly lower than for the US general population (Fig. 3b). The most affected domain in the total study population was *Physical function* (mean 39.5), followed by *Pain interference* (mean 58.4). Additionally, the study participants reported more symptoms of anxiety and fatigue compared to the general population, as well as a reduced ability to participate in social roles and activities.

#### Achondroplasia/hypochondroplasia vs. other skeletal dysplasia conditions

Compared to the achondroplasia/hypochondroplasia group, participants with other skeletal dysplasia conditions reported lower scores in all domains for the SF-36/RAND-36 except for mental health, as well as lower scores in the physical and mental health component scores (PCS and MCS). The highest differences were observed in the domains *Role limitations due to physical function, Physical functioning*, and *Role limitations due to mental health* (Table 5 and Fig. 3c).

Consistent with the findings in the SF-36/RAND-36, participants with other skeletal dysplasia conditions reported reduced HRQOL compared to the achondroplasia/hypochondroplasia group as measured by the physical and mental component scores in the PROMIS-29 (Table 5). On the domain level, all domains except for *Depression* and *Anxiety* were significantly lower in participants with other skeletal dysplasia conditions (Fig. 3d). *Physical function* was the domain with the lowest scores in both groups, while the largest differences were observed in the domains *Fatigue* and *Pain interference*.

## Discussion

This study aimed to investigate the prevalence and symptom severity of mental health conditions, physical functioning and HRQOL among short-statured adults with a skeletal dysplasia.

A history of psychiatric diagnosis was present in more than one third of the total study population, where depression or anxiety were the most prevalent. The prevalence of current clinically significant symptoms of depression and anxiety was considerably higher in the skeletal dysplasia study population compared to the US general population. The study population also reported a high burden of pain and more difficulties in performing daily activities. HRQOL, as assessed by the SF-36/RAND-36 and PROMIS-29, was lower in the skeletal dysplasia study population compared to the general population for all physical and mental health domains.

Within the skeletal dysplasia study population, individuals with other skeletal dysplasia conditions demonstrated overall lower scores across all parameters for both physical and mental health as well as more pain, reduced physical functioning, and lower HRQOL, compared to participants with achondroplasia or hypochondroplasia.

In this study, 23% of the total study population reported current and clinically significant symptoms of depression (PHQ-8 score ≥ 10), while current clinically significant anxiety symptoms (GAD-7 ≥ 10) were reported by 13%. The percentages of individuals who screened positive for current, clinically significant depression and/or anxiety is considerably higher than in the US general population, where the prevalence of moderate and severe depression and anxiety are 7% and 6%, respectively, based on data from the National Health Interview Survey [32, 33].

However, the high prevalence of depression and anxiety in our study population is consistent with a prior (2019) US study, reporting on 336 adults with a skeletal dysplasia (of whom more than half had achondroplasia) [6]. In that study, conducted by Jennings et al., 16% reported clinically significant symptoms of depression and 17% of anxiety, as measured by PHQ-8 and GAD-7 [6]. Furthermore, 29% in the US study reported a prior diagnosis of depression and 25% a prior diagnosis of anxiety [6]. These numbers are in line with our findings, where 23% reported a history of anxiety and 22% a history of depression. The high prevalence of depression is also consistent with a prior study conducted in the UK in 2010 by Shakespeare et al. They found that 31% of their study population of adults with a skeletal dysplasia (more than half with achondroplasia) reported a previous history of depression [8]. Taken together, these findings suggest an elevated risk for mental health issues, and in particular depression and anxiety, in adults with a skeletal dysplasia.

From previous studies, we know that living with a short stature might carry a risk of experiencing social stigma, such as staring, teasing, and discrimination, both in children and adults [8, 35]. Based on focus groups and interviews with Spanish and US adolescents with achondroplasia, Shediac et al. found that many participants reported unwanted attention in public, including staring and receiving comments from strangers, as well as prejudice and bullying [36]. These social experiences had negative emotional impacts, leading to reduced self-esteem and feelings of frustration and anger [36]. Their findings are consistent with a qualitative study of Norwegian adults with short stature by Schanke and Thorsen, published in 2014 [37]. The study participants reported experiences of feeling lonely, being treated as “the other,” encountering difficulties in attracting a partner, as well as being subject to bullying. Collectively, these negative social experiences constitute considerable vulnerability factors for developing mental health issues [38].

More than half of those with a prior diagnosis of depression, and about one third of those with a previous history of anxiety, reported current clinically significant symptom levels of depression and anxiety on the PHQ-8 or GAD-7. This difference might be due to treatment, spontaneous remission, or to changes in life circumstances over time, but also underlines the importance of regular assessment of mental health as part of the regular medical check-up across the lifespan.

The higher prevalence of mental health issues (depression and anxiety) among the UK respondents could partly be explained by their overall lower education and income of the responding participants. It is known that lower socioeconomic status is associated with higher prevalence of mental health disorders in the general population [39, 40].

Consistent with previous studies, the physical health measures in the skeletal dysplasia study population were lower than for the general US population for all health domains, and for the physical health component score (PCS). The high pain prevalence and pain intensity (mean 6.0) in our study population are also consistent with previous studies [4–7] and was mainly related to orthopaedic and neurologic complications. This is also reflected by the high surgical burden in the study population, with a mean of more than 7 surgical procedures per participant, ranging from 0 up to 36. The high surgical burden is consistent with previous studies conducted on children and adults with achondroplasia [41, 42].

Regarding the ability to perform activities of daily living (ADL), the mean HAQ score, as well as all eight category scores, were considerably higher in the study population compared to norms for the general population, indicating more difficulties in performing the tasks. In a Finnish study of 1530 participants, the estimated general population mean of HAQ was 0.25 [43], compared to 0.85 in our study population. Indeed, the results in our study population were in the same range as reported in a study on adults with rheumatoid arthritis (mean score 0.91) [44].

Notably, compared to study participants with achondroplasia/hypochondroplasia, participants with other skeletal dysplasia conditions reported overall lower scores across almost all parameters for both physical and mental health, more pain site locations, higher pain intensity, higher degree of clinically significant pain catastrophizing, and lower scores for ADL and HRQOL (both for SF-36/RAND-36 and PROMIS-29). It is well known from studies conducted in the general population that the degree of physical disability and pain may have a negative impact on mental health [45, 46]. In this study, individuals with other skeletal dysplasia conditions overall reported higher prevalence of pain in the upper and lower extremities, neck and upper back. They also reported higher prevalence of spine surgery, joint replacement surgery as well as other surgery related to the upper and lower extremities. This corresponds to the findings of more affected ADL in the group of other skeletal dysplasia conditions, particularly in the domains “walking”, “reach”, “grip” and “activities”. Moreover, previous studies in people with skeletal dysplasia conditions have found a strong relationship between pain intensity and psychological functioning [6, 7]. This might explain the higher prevalence of mental health issues in the group of other skeletal dysplasia conditions observed in this study. Finally, the vulnerability factors associated with living with a rare disorder might be even more pronounced in individuals with very rare skeletal conditions, including lack of knowledge of the condition by health care professionals and others, and feeling alone with the condition [47].

We included both SF-36/RAND-36 and PROMIS-29 in this study. The SF-36/RAND-36 has been widely studied and cited in the skeletal dysplasia HRQOL literature [48]. However, more recently the PROMIS-29 has become more commonly used in different patient populations [49–51]. We included both to be able to compare our results to previous published literature on skeletal dysplasia conditions. Furthermore, the normative sample for PROMIS-29 is based on more recent studies [52].

### Strengths and limitations

There are several limitations to this study. Although we used validated self-report instruments to measure the outcomes, the use of self-report measures gives a risk of response bias. The relatively small study sample also give a risk of selection bias. The majority of the US and UK study population was recruited from clinical practice. This may impose a risk of selection bias, as individuals seeking medical care may exhibit a higher degree of health challenges. There was a considerable difference in education level and annual income between the respondents from the three study centres. This may affect the reported prevalence rates of mental health conditions, and caution should therefore be exercised in drawing conclusions from the observed differences in mental health prevalence rates across the three countries. However, despite these limitations, the key findings in this study both on mental and physical health, pain, physical functioning, and HRQOL, are consistent with previous studies, indicating that our results might be representative for the population of individuals with a skeletal dysplasia. Finally, both in the achondroplasia/hypochondroplasia group, and even more in the group of other skeletal dysplasia conditions, there was a considerable heterogeneity, which may mask condition-specific outcomes and preclude further comparison across these diagnostic subgroups. Lastly, the use of US population norms may not be representative to the Norwegian and British general population.

## Conclusion

In this study, we found reduced physical and mental health scores in adults with skeletal dysplasia conditions compared to the general population. Symptoms of depression and anxiety were prevalent, including a high prevalence and burden of pain, reduced ability to perform daily activities, and impaired HRQOL. The causes are likely multifactorial, consisting of psychosocial and socioeconomic difficulties associated with short stature as well as a high level of chronic pain, medical complications, and a high surgical burden. Participants with skeletal dysplasia conditions other than achondroplasia/hypochondroplasia reported lower physical and mental health scores, higher burden of pain, and more reduced physical functioning and more reduced HRQOL.

Collectively, the high prevalence of mental health issues, pain, and reduced physical functioning in individuals with a skeletal dysplasia highlights the importance of including an assessment of these issues at every medical follow-up across the lifespan in these patients [53].

New drug treatment options have recently been approved for infants and children with achondroplasia [54]. Clinical trials are also ongoing in children with hypochondroplasia [55], and the rare disease community is advocating for better care and management which will hopefully lead to new management and treatment options in the future. One of the challenges of assessing the effectiveness of new treatment is to identify correct and useful outcome measures. Growth velocity and height are objective and easily measurable data. However, our findings underline the importance of also including evaluation of HRQOL, including mental health, physical functioning, and pain as long-term outcomes to assess the clinical utility of possible new interventions.

## Data Availability

The data that support the findings of this study are not publicly available due to ethical and confidentiality concerns. The data from the current study are available from the corresponding author on reasonable request.

## Abbreviations

ACH: Achondroplasia
HCH: Hypochondroplasia
HRQOL: Health-related quality of life
MCS: Mental health component score
OSD: Other skeletal dysplasia conditions
PCS: Physical health component score
